# Strategies to Promote Long-Distance Optic Nerve Regeneration

**DOI:** 10.3389/fncel.2020.00119

**Published:** 2020-05-14

**Authors:** Shu-Guang Yang, Chang-Ping Li, Xue-Qi Peng, Zhao-Qian Teng, Chang-Mei Liu, Feng-Quan Zhou

**Affiliations:** ^1^State Key Laboratory of Reproductive Biology, Institute of Zoology, Chinese Academy of Sciences, Beijing, China; ^2^Department of Orthopaedic Surgery, The Johns Hopkins University School of Medicine, Baltimore, MD, United States; ^3^The Solomon H. Snyder Department of Neuroscience, The Johns Hopkins University School of Medicine, Baltimore, MD, United States

**Keywords:** optic nerve, axon regeneration, retinal ganglion cells, functional recovery, glaucoma

## Abstract

Mammalian retinal ganglion cells (RGCs) in the central nervous system (CNS) often die after optic nerve injury and surviving RGCs fail to regenerate their axons, eventually resulting in irreversible vision loss. Manipulation of a diverse group of genes can significantly boost optic nerve regeneration of mature RGCs by reactivating developmental-like growth programs or suppressing growth inhibitory pathways. By injury of the vision pathway near their brain targets, a few studies have shown that regenerated RGC axons could form functional synapses with targeted neurons but exhibited poor neural conduction or partial functional recovery. Therefore, the functional restoration of eye-to-brain pathways remains a greatly challenging issue. Here, we review recent advances in long-distance optic nerve regeneration and the subsequent reconnecting to central targets. By summarizing our current strategies for promoting functional recovery, we hope to provide potential insights into future exploration in vision reformation after neural injuries.

## Introduction

Retinal ganglion cells (RGCs) relay visual related information from the eye to the brain through their axons, which collectively form the optic nerve (Laha et al., 2017). Optic nerve injuries induced by trauma, glaucoma or neurodegenerative diseases often result in loss of visual functions and eventually blindness. Strategies promoting RGC survival and optic nerve regeneration are actively pursued to repair neural injury and restore visual function. As central nervous system (CNS) neurons, mature RGCs have greatly reduced intrinsic capacity to regenerate their axons after traumatic injuries or neurodegeneration, eventually leading to loss of vision (Chun and Cestari, 2017; Laha et al., 2017). Also, contrary to neurons in the peripheral nervous system (PNS), various extrinsic inhibitory molecules act to limit axon regeneration in the CNS, including the spinal cord and optic nerves (Geoffroy and Zheng, 2014). Previous studies showed that removing extracellular inhibitory factors, such as Nogo and its receptors, could induce mild optic nerve regeneration (Fischer et al., 2004; Su et al., 2008, 2009; Dickendesher et al., 2012). In contrast, deleting phosphatase and tensin homolog (Pten) in RGCs, which boosted the intrinsic axon regeneration capacity, promoted robust optic nerve regeneration, indicating promising new avenues for enhancing CNS axon regeneration (Park et al., 2008). During the past decade, manipulation of several genes in RGCs has been shown to significantly boost the intrinsic axon regeneration capacity of mature RGCs, such as *Klf4/9* (Moore et al., 2009; Apara et al., 2017), *Socs3* (Smith et al., 2009), *B-RAF* (O’Donovan et al., 2014), *c-myc* (Belin et al., 2015), *GSK3β* (Guo et al., 2016; Miao et al., 2016), *Lin28* (Wang et al., 2018), and *P53* (Ma et al., 2019). Although, these genes have been shown to regulate optic nerve regeneration, almost none of them alone could be manipulated to induce long-distance axon regrowth *in vivo*. To solve this problem, combinatorial strategies have been tried to extend the lengths of regenerative axons. Indeed, the synergistic or additive effects of multiple independent pathways on RGC axon regeneration were more dramatic and sustainable, such as Zymosan/cAMP/Pten-deletion (Kurimoto et al., 2010), CNTF/Pten-deletion/Socs3-deletion (Sun et al., 2011), c-Myc/CNTF/Pten-deletion/Socs3-deletion (Belin et al., 2015), and Lin28/Pten-deletion (Wang et al., 2018). In a few studies, it was reported that regenerating RGC axons could reach long distance, crossing the optic chiasm and entering the brain (Kurimoto et al., 2010; Sun et al., 2011; de Lima et al., 2012; Lim et al., 2016).

The next step after long-distance optic nerve regeneration should be exploring how visual function could be restored with proper axon guidance, synaptogenesis, and neural activity transmission. To date, *de novo* optic nerve regeneration across the chiasm appears to be the bottleneck for regenerating RGC axons to enter the brain. Therefore, only a few studies using combinatory approaches have reported very limited reconnection between injured optic nerve axons and their targets in the brain, such as the suprachiasmatic nucleus (SCN), the lateral geniculate nucleus (LGN), the superior colliculus (SC), and other visual areas with either longer period after the injury (de Lima et al., 2012; Bei et al., 2016; Lim et al., 2016) or performing the injury at the pre-chiasm (Li et al., 2015) or optic tract (Bei et al., 2016). Although further confirmation of these studies is still needed, the results provided some proof-in-principle evidence that visual function recovery is possible after optic nerve injury if each step of axon regrowth, guidance, synaptogenesis, and remyelination could be achieved. Here, we review recent progress in achieving the reconnection of the eye-to-brain pathways and discuss potential future strategies for rewiring the visual circuits after optic nerve injuries.

## Long-Distance Axon Regeneration Can Be Achieved *via* Combinatory Manipulation of Multiple Genes/Pathways

**Figure 1:**
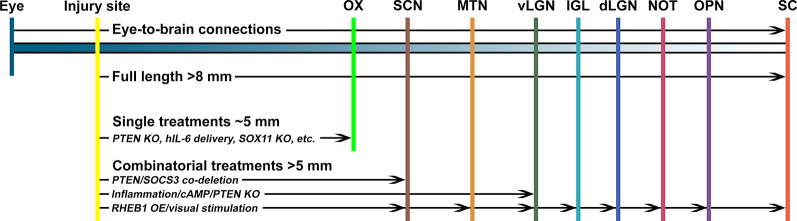
The promoting capacity of known treatments on optic nerve regeneration *in vivo*. To regain visual function, regenerating optic nerve axons need to cross the optic chiasm (OX) and reach specific nuclei in the brain, including the suprachiasmatic nucleus (SCN), medial terminal nucleus (MTN), thalamic ventral or dorsal lateral geniculate nucleus (vLGN, dLGN), intergeniculate leaflet (IGL), the nucleus of the optic tract (NOT), olivary pretectal nucleus (OPN), and superior colliculus (SC). Manipulation of a single factor, such as Pten knockout (PTEN KO), IL6 expression, or Sox11 overexpression (SOX11 OE), is unlikely to enhance optic nerve regeneration to reach the OX. However, combinatorial approaches with multiple factors can induce longer distance axon regeneration to reach and cross the OX. In a few cases, it was reported that a combination of multiple factors, such as Pten/Socs3 co-deletion, inflammation/cAMP/Pten knockout, or Rheb1 overexpression/visual stimulation, could enhance optic nerve regeneration to reconnect with selected brain nuclei.

To restore vision after optic nerve injury, injured axons must regenerate the full length of the eye-to-brain pathways, a distance of more than 8 mm from the injury site to LGN and SC in mice (Figure 1). Long-distance axon regeneration, as the first step of the eye-to-brain reconnection, is crucial in the restoration of visual function following optic nerve injury. To date, conditional knocking out Pten alone in RGCs led to probably the longest optic nerve regeneration at 2 weeks after injury (up to 3 mm distal to the lesion site; Park et al., 2008). Manipulations of other genes, as listed in Table 1, have been shown to promote modest regeneration of RGC axons reaching the medium region of the optic nerve after injury (Table 1). In addition to manipulation of gene expression in RGCs, the non-RGC-mediated release of CNTF (Leaver et al., 2006), oncomodulin in response to inflammation (Yin et al., 2006), or amacrine-specific Lin28-mediated IGF1 potentiation (Zhang et al., 2019), have all been shown to stimulate optic nerve regeneration, either alone or together with other factors. Moreover, an elevated level of zinc in amacrine cells upon optic nerve injury has been shown to contribute to RGC cell death and failed regeneration by slowly transferring into RGCs (Li et al., 2017). As a result, the zinc transporter ZnT-3 (encoded by gene slc30a3) knockout enhanced RGC survival and regeneration. Furthermore, an increased level of cAMP has been shown to enhance oncomodulin-induced optic nerve regeneration (Kurimoto et al., 2010). Lastly, a subtype of RGCs have shown to produce a secreted phosphorylated glycoprotein, osteopontin (OPN), which acts together with IGF1 or BDNF, to enhance optic nerve regeneration (Duan et al., 2015).

**Table 1:** Genetic manipulation for promoting optic nerve regeneration in mice.

Gene	Modulation	Phenotype		Reference
*Pten*	Deletion	Promoted axon regeneration	Promoted neuronal survival	Park et al. (2008)
*Socs3*	Deletion	Promoted axon regeneration	Promoted neuronal survival	Smith et al. (2009)
*Klf4*	Deletion	Promoted axon regeneration		Moore et al. (2009)
*Thbs1*	Overexpression	Promoted axon regeneration		Bray et al. (2019)
*Mettl14*	Knockdown	Attenuated axon regeneration	Attenuated neuronal survival	Weng et al. (2018)
*Mdm4*	Deletion	Promoted axon regeneration		Joshi et al. (2015)
*Sox11*	Overexpression	Promoted axon regeneration	Killed α-RGC	Norsworthy et al. (2017)
*Lin28*	Overexpression	Promoted axon regeneration		Wang et al. (2018)
*Dclk2*	Overexpression	Promoted axon regeneration	Promoted neuronal survival	Nawabi et al. (2015)
*Armcx1*	Overexpression	Promoted axon regeneration	Promoted neuronal survival	Cartoni et al. (2016)
*Mlp*	Overexpression	Promoted axon regeneration		Levin et al. (2019)
*p53*	Overexpression	Promoted axon regeneration		Ma et al. (2019)
*Tet1*	Knockdown	Attenuated axon regeneration		Weng et al. (2017)
*Socs4*	Knockdown	Promoted axon regeneration		Sekine et al. (2018)
*Rab27b*	Deletion	Promoted axon regeneration		Sekine et al. (2018)
*Rheb1*	Overexpression	Promoted axon regeneration		Lim et al. (2016)
*Cntf*	Overexpression	Promoted axon regeneration	Promoted neuronal survival	Pernet et al. (2013a)
*Stat3*	Activation	Promoted axon regeneration		Luo et al. (2016)
*Opn4*	Overexpression	Promoted axon regeneration		Li et al. (2016)
*Akt*	Activation	Promoted axon regeneration	Promoted neuronal survival	Guo et al. (2016)
*Gsk3b*	Deletion	Promoted axon regeneration		Leibinger et al. (2017)
*Eif2b5*	Activation	Promoted axon regeneration		Guo et al. (2016)
*hIL-6*	Expression	Promoted axon regeneration	Promoted neuronal survival	Leibinger et al. (2016)
*c-myc*	Overexpression	Promoted axon regeneration	Promoted neuronal survival	Belin et al. (2015)
*Dlk*	Deletion	Attenuated axon regeneration		Watkins et al. (2013)
*S6k1*	Activation	Promoted axon regeneration		Yang et al. (2014)
*Eif4ebp*	Deletion		Promoted neuronal survival	Yang et al. (2014)
*Rtca*	Deletion	Promoted axon regeneration		Song et al. (2015)
*Klf9*	Knockdown	Promoted axon regeneration	Promoted neuronal survival	Apara et al. (2017)
*slc30a3*	Deletion	Promoted axon regeneration	Promoted neuronal survival	Li et al. (2017)
*Hdac5*	Dephosphorylation	Promoted axon regeneration	Promoted neuronal survival	Pita-Thomas et al. (2019)
*Hsp70*	Overexpression		Promoted neuronal survival	Kwong et al. (2015)
*Dusp14*	Deletion		Promoted neuronal survival	Galvao et al. (2018)
*Wnt3a*	Application	Promoted axon regeneration	Promoted neuronal survival	Patel et al. (2017)
*mir-135*	Mimics	Promoted axon regeneration		van Battum et al. (2018)
*Crmp2*	Activation	Promoted axon regeneration		Leibinger et al. (2017)
*Pedf34*	Application	Promoted axon regeneration	Promoted neuronal survival	Vigneswara et al. (2015)
*Rkip*	Overexpression	Promoted axon regeneration	Promoted neuronal survival	Wei et al. (2015)
*B-raf*	Overexpression	Promoted axon regeneration		O’Donovan et al. (2014)
*Nrn1*	Overexpression	Promoted axon regeneration	Promoted neuronal survival	Sharma et al. (2015)
*Ddit3*	Deletion		Promoted neuronal survival	Hu et al. (2012)
*Xbp1*	Overexpression		Promoted neuronal survival	Hu et al. (2012)
*p75ntr*	Suppression	Promoted axon regeneration		Uesugi et al. (2013)
*Rtn4*	Overexpression	Enhanced axonal growth	Attenuated neuronal survival	Pernet et al. (2012)
*Arg2*	Deletion	Enhanced axonal sprouting	Promoted neuronal survival	Xu et al. (2018)
*NgR*	Deletion	Promoted axon regeneration		Su et al. (2009)
*Lpin1*	Knockdown	Promoted axon regeneration		Yang et al. (2020)
*Lgals3*	Deletion		Promoted neuronal survival	Abreu et al. (2017)
*Trif*	Deletion	Promoted axon regeneration	Promoted neuronal survival	Lin et al. (2012)
*NFκB*	Activation		Promoted neuronal survival	Dvoriantchikova et al. (2016)
*Crtac1b*	Overexpression	Promoted axon regeneration		Hirokawa et al. (2017)
*Efnb3*	Deletion	Promoted axon regeneration		Duffy et al. (2012)
*Bag1*	Overexpression	Promoted axon regeneration	Promoted neuronal survival	Planchamp et al. (2008)
*Shp*	Knockdown	Promoted axon regeneration		Fujita et al. (2011)
*Dock3*	Overexpression	Promoted axon regeneration		Namekata et al. (2010)
*Ucn*	Overexpression	Promoted axon regeneration	Promoted neuronal survival	Tran et al. (2019)
*Timp2*	Overexpression	Promoted axon regeneration	Promoted neuronal survival	Tran et al. (2019)
*Crhbp*	Deletion	Promoted axon regeneration	Promoted neuronal survival	Tran et al. (2019)
*Mmp9*	Deletion	Promoted axon regeneration	Promoted neuronal survival	Tran et al. (2019)

Although several approaches have been shown to promote substantial regeneration *in vivo*, the regrowth of sufficient numbers of axons through the entire optic pathway remains a major challenge. Thus, recent advances in RGC axon regeneration not only focused on identifying novel genes and pathways, but also revealed that combinatorial treatments with distinct underlying mechanisms resulted in additive or even synergistic effects (Table 2). Up to date, Pten deletion, together with manipulation of other RGC genes or extracellular factors, have been the dominant combinatory strategy for promoting long-distance optic nerve regeneration. For instance, Socs3 deletion (Sun et al., 2011), B-RAF activation (O’Donovan et al., 2014), c-Myc overexpression (Belin et al., 2015), DCLK2 overexpression (Nawabi et al., 2015), hIL-6 expression (Leibinger et al., 2016), STAT3/MEK activation (Luo et al., 2016), zinc chelation (Li et al., 2017), or Lin28 overexpression (Wang et al., 2018), has each been shown to have synergistic/additive effects with Pten deletion on optic nerve regeneration. Also, extracellular factors described above have all been combined with genetic manipulation of RGCs, such as cAMP/oncomodulin/Pten-deletion (Kurimoto et al., 2010), CNTF/Pten-deletion/Socs3-deletion (Sun et al., 2011). Furthermore, the additive or synergistic effects of other combinatorial strategies have also been shown, such as Klf9 knockdown combined with Zinc chelation (Trakhtenberg et al., 2018) and constitutively active CRMP2 combined with enhanced GSK3 activity (Leibinger et al., 2017). Such combinatory effects are believed to be due to different signaling pathways downstream of these genes and factors. Based on previous studies, several signaling pathways have been shown to play important roles in transducing the promoting effects of these genes/factors. For instance, the growth-factor related GSK3/mTOR signaling is activated downstream of Pten deletion (Park et al., 2008; Leibinger et al., 2019), osteopontin (Duan et al., 2015), melanopsin (Li et al., 2016), Akt (Guo et al., 2016; Miao et al., 2016), or Lin28 (Wang et al., 2018). Also, the MAPK pathway downstream of B-RAF (O’Donovan et al., 2014) or MEK (Luo et al., 2016) was involved. Moreover, the Jak-Stat cytokine signaling could be activated downstream of CNTF (Müller et al., 2007), Socs3 deletion (Smith et al., 2009), or Klf4 deletion (Qin et al., 2013). Furthermore, recent studies have revealed several novel signaling pathways functioning to promote optic nerve regeneration, such as the phosphatidic acid phosphatase (PAP) Lipin1 that induced regeneration through regulating glycerolipid metabolism (Yang et al., 2020), thrombospondin-1 that bound to syndecan to promote optic nerve regeneration (Bray et al., 2019), and the actin cross-linker muscle LIM protein (Levin et al., 2019). It is worth noting that many optic nerve regeneration regulatory genes can simultaneously regulate multiple downstream pathways and the downstream signaling pathways have also been shown to crosstalk with each other.

**Table 2:** Long-distance optic nerve regeneration in mice.

Manipulation	Time	Assessment of regenerating axons	Reference
*Pten* deletion	4 weeks	Until the optic chiasm	Park et al. (2008)
Hyper-IL-6 expression	6 weeks	Within the optic chiasm and the contralateral optic nerve	Leibinger et al. (2016)
SOX11 overexpression	4 weeks	>4 mm	Norsworthy et al. (2017)
KLF9 knockdown	2 weeks	Within the optic chiasm and the contralateral side	Apara et al. (2017)
Glia-targeting AAV.DH-CNTF	8 weeks	Until the optic chiasm	Pernet et al. (2013a)
B-RAF expression/*Pten* deletion	2 weeks	>3.5 mm	O’Donovan et al. (2014)
DCLK2 overexpression/*Pten* deletion	2 weeks	Until the optic chiasm	Nawabi et al. (2015)
*Pten* and *Socs3* co-deletion (Pre-chiasm lesion)	8 weeks	Within the core region of SCN and functionally active synaptic connections	Li et al. (2015)
RHEB1 overexpression/Biased visual stimulation	3 weeks	Within multiple subcortical visual targets and partial recovery of visual function	Lim et al. (2016)
Zinc chelation/*Pten* deletion	12 weeks	Across the optic chiasm	Li et al. (2017)
SOX11 overexpression/*Pten* deletion	7 weeks	Across the optic chiasm and within the optic tract	Norsworthy et al. (2017)
*Bax* knockout/Delayed CNTF overexpression	8 + 8 weeks	Within the optic chiasm and the SCN	Yungher et al. (2017)
Zinc chelation/*Klf9* knockdown	6 weeks	Within the optic chiasm and the ipsilateral optic tract	Trakhtenberg et al. (2018)
Zymosan/cAMP/*Pten* deletion	6 weeks	Within the optic chiasm and the LGN	Kurimoto et al. (2010)
	10–12 weeks	Within the major visual targets (the SCN, OPT, MTN, LGN, and SC) and partial recovery of visual function	de Lima et al. (2012)
	10–12 weeks	Within the optic tract and the SCN (3D projection)	Luo et al. (2013)
	12 weeks	Within the contralateral SCN, dLGN and SC	Goulart et al. (2018)
*Pten* and *Socs3* co-deletion/CNTF overexpression	4 weeks	Across the optic chiasm and within the SCN	Sun et al. (2011)
*Pten* knockdown/cAMP/ CNTF overexpression	4–6 weeks	Within the optic tract and the SCN (3D projection)	Yungher et al. (2015)
STAT3 and MEK co-activation/*Pten* deletion	10 weeks	Across the optic chiasm and within the brain (3D projection)	Luo et al. (2016)
CRMP2 and GSK3 co-activation/Lens injury	3 weeks	Until the optic chiasm	Leibinger et al. (2017)
c-Myc and CNTF co-overexpression/*Pten* and *Socs3* co-deletion	4 weeks	Across the optic chiasm and within the optic tract (Whole-mount analysis)	Belin et al. (2015)

Collectively, it is well recognized that the combination of molecules with different downstream pathways would result in longer distance axon regeneration. Thus, identifying new factors with novel signaling pathways, especially factors that can regulate multiple pathways, should still be one of the focused areas in the field. Besides, exploring the optimal combination of multiple factors based on their regulatory mechanisms is also very important. Lastly, it should be noted that the majority (>80%) of RGCs die a few weeks after optic nerve injury (Wang et al., 2018; Tran et al., 2019), making RGC survival a major obstacle for a sufficient number of regenerating axons necessary for visual function recovery. Intriguingly, several subtypes of RGCs have been shown to differ in their ability to survive or regenerate axons upon optic nerve injury (Duan et al., 2015; Norsworthy et al., 2017; Bray et al., 2019). Recently, a systematic study by using single-cell RNA-seq revealed the selective vulnerability of RGC subtypes following axonal injury and provided evidence that type-specific neuroprotective strategies could be critical for intervention (Tran et al., 2019). Thus, genes/pathways that act to protect RGCs or subtypes from cell death are equally important to be identified.

## Proper Guidance of Regenerating Axons Passing The Optic Chiasm and Entering The Brain

Although the combinatorial approaches after optic nerve injury could result in substantial long-distance optic nerve regeneration, the optic chiasm is emerging to be the major obstacle for regenerating axons to enter the brain and reach their original targeted nuclei. By analyzing axon regeneration at later time points after the nerve injury (i.e., 4–10 weeks), in several studies using combinatory approaches regenerating axons could reach and even cross the optic chiasm. For example, when Pten/Socs3 double knockout was combined with CNTF, 4 weeks (Sun et al., 2011) or 10 weeks (Luo et al., 2013) after nerve injury many regenerating axons could reach the proximal end of the chiasm but stopped growing. When c-Myc expression was added into the combination, more axons crossed the chiasm and grew into the optic tracts (Belin et al., 2015). Similarly, other combinatory approaches, such as cAMP/Zymosan/Pten-deletion (de Lima et al., 2012; Luo et al., 2013), Lipin1-deletion/CNTF (Yang et al., 2020), and Pten-deletion/MEK/STAT3 (Luo et al., 2016), could also induce optic nerve regeneration near or cross the optic chiasm.

One interesting observation from some of these studies was that many regenerating axons were turned back at the chiasm or derailed away from the optic tract after crossing the chiasm. This observation suggests that optic chiasm presents an inhibitory boundary and misguidance of axons occurs in the optic tract. Indeed, as aforementioned, deleting Nogo and its receptors in the visual system resulted in mild optic nerve regeneration, indicating the inhibitory nature of the mature visual system. In an early study (Pernet et al., 2013a), by using tissue clearing approach and confocal imaging of the whole mount optic nerve, the study found that many regenerating axons induced by CNTF showed irregular trajectories, with many U-turns within the optic nerve. In a later study from the same lab (Pernet et al., 2013b), regenerating RGC axons induced by active STAT3 also showed wandering trajectories with frequent U-turns. However, when the optic nerves were additionally treated with the Rho kinase inhibitor Y27632, the Stat3-induced regenerating axons became straighter and the U-rate was markedly reduced. Because Rho kinase inhibitor is well known to antagonize the inhibitory effects of myelin-based inhibitor (i.e., Nogo, MAG), these results confirmed the presence of extracellular inhibitory molecules in the optic nerve. In support, by using tissue clearing and advanced light-sheet fluorescence microscopy (LSFM), three studies (Luo et al., 2013; Yungher et al., 2015; Bray et al., 2017) performed high-resolution 3D imaging and detailed axonal morphological analyses at the single axon level. Specifically, the results found that within the optic nerve most regenerating axons had a meandering path and many of them made sharp turns. For axons reaching the chiasm, some axons turned back at the chiasm and enter the opposite uninjured optic nerve, confirming chiasm as an inhibitory barrier. For axons that managed to cross the chiasm, more axons were observed in the ipsilateral optic tract than in the contralateral tract, indicating axon misguidance. A few axons were identified in the SCN located directly above the chiasm and no axon was observed in the more distant visual targets, LGN or SC. Consistent with these findings, our recent study using tissue clearing and 3D imaging (Wang et al., 2018) showed that Lin28 overexpression-induced regenerating RGC axons also showed many U-turns within the optic nerve, which was reduced when Lin28 expression was combined with Pten knockdown. Moreover, our latest study (Wang et al., 2020) showed that knocking out myosin IIA/B in RGCs alone resulted in significant optic nerve regeneration. Interestingly, when axon trajectories were examined with tissue clearing and 3D imaging, in wild type mice the automatically regenerated short axons followed wandering trajectories with many U-turns, whereas in myosin IIA/B knockout mice the regenerating axons were much straighter with greatly reduced U-turns. More importantly, combining Lin28 overexpression with myosin IIA/B knockout led to long-distance optic nerve regeneration in 2 weeks after the nerve injury (up to 4.5 mm from the injury site). Because our previous study (Hur et al., 2011) showed that deleting myosin IIA/B allowed axon growth over two major inhibitory substrates, myelin debris, and CSPGs, the new results provided evidence that overcoming the inhibitory signals in the optic nerve was a promising approach for more efficient long-distance optic nerve regeneration.

Lastly, to better examine how regenerating axons behaved at the optic chiasm, one study (Li et al., 2015) used a pre-chiasm injury model, in which regenerating axons only needed to grow a short distance to reach the chiasm. The study demonstrated that at 4 weeks after the nerve injury, many axons entered and passed the chiasm, most of which occupied the ipsilateral side of the hypothalamus, including the SCN. At 8 weeks, more axons were found within the SCN. One important finding was that even at 4 months after the nerve injury, almost no regenerating axons reached brain targets further away from the optic chiasm, such as the OPN and the SC. This study further confirmed that without proper guidance cues, it is difficult for regenerating axons to reinnervate deeper brain targets.

In summary, these studies suggested that proper axon guidance mechanisms are necessary for regenerating RGC axons to reach their original targeted nuclei in the brain and the subsequent visual functional recovery. New experimental techniques, such as tissue clearing approaches (3DISCO, CLARITY, etc.) and advanced 3D imaging systems (multiphoton microscopy, LSFM, fMOST, etc.), are emerging to be useful tools for detailed analysis of axon trajectory in the brain.

## Axon Regeneration from The Eye to The Brain Rescuing Partial Visual Behaviors

After regenerating axons reach their original brain targets, the next challenge is to achieve new synaptogenesis, remyelination, and the subsequent functional restoration of vision. To date, only a few studies have reported functional reconnection with brain nuclei after optic nerve crush. An early study showed that the combination of Pten deletion with Zymosan and cAMP resulted in long-distance axon regeneration crossing the optic chiasm and into brain structures, including SCN, dLGN, and SC, 10 weeks after the optic nerve injury. Histological evidence demonstrated that regenerating axon terminals appeared to form synapses within the targeted nuclei. Consequently, several innate visual behaviors were partially restored, such as depth perception, optomotor response, and circadian activity patterns (de Lima et al., 2012). In a later study in which the same combinatory approach was used (Luo et al., 2013), whole-mount tissue clearing and 3D imaging analysis with LSFM showed that regenerating axons were indeed observed into and beyond the optic chiasm. However, in contrast to the above study (de Lima et al., 2012), only some axons were observed reaching the SCN and no regenerating axons were found beyond the SCN. Many axons were observed in places that were not associated with the optic pathway, indicating axon misguidance. In the same study, Socs3/Pten double knockout, together with CNTF, were used to induce long-distance axon regeneration. Similarly, regenerating axons could reach and cross the optic chiasm and no axons could be found beyond the SCN. To rule out the possibility that the tissue clearing procedure might bleach the CTB tracer signal, regular coronal brain sections were examined. Similarly, CTB labeled regenerating axons were mainly found in the hypothalamus area, including the SCN, and no axons were identified in the more distant targets, LGN and SC.

In a recent study, by enhancing RGC neural activity and activating the mTOR signaling (Rheb1 overexpression) long-distance axon regeneration and correct pathfinding into all major visual targets were observed as early as 3 weeks after the optic nerve injury (Lim et al., 2016). Specifically, in mice receiving visual stimulation and Rheb1 overexpression treatment, newly formed connections could partially restore optomotor response by optokinetic reflex analysis (OKR), whereas failed to rescue pupil response, depth perception, and visual fear response (Lim et al., 2016). The study suggested that such partial functional recovery might reflect defects in synapse formation, insufficient numbers of regenerating axons, and/or low precision of within-target pathfinding. Together, to date, there are only very few studies (de Lima et al., 2012; Lim et al., 2016) reported long-distance optic nerve regeneration with partial visual function recovery, among which one study could not be repeated in a different study (Luo et al., 2013). Thus, additional studies are necessary to confirm if after intraorbital optic nerve injury regenerating RGC axons can be correctly guided to reach their original innervating targets in the brain.

Despite significant advances in promoting RGC axons beyond the optic chiasm into the brain after optic nerve injury, it is difficult to explore axon pathfinding across the chiasm and the subsequent target reinnervation due to the limited number of regenerating axons reaching the brain. To solve such a problem, as mentioned above in one study (Li et al., 2015) pre-chiasm optic nerve injury was performed so that more axons could reach and cross the optic chiasm. In this study, serial sections revealed that after a longer period more regenerating axons could be identified at the core region of SCN. By focusing on the SCN, the study used antibodies against both the pre-synaptic marker vesicular glutamate transporter 2 (VgluT2) and the postsynaptic density marker Homer1 to label new synapse formation. Many triple labeled (CTB, VgluT2, Homer1) dots were identified, indicating excitatory synaptic sites on regenerating CTB positive regenerating axons. Additional experiments using pseudo-rabies virus encoding GFP provided evidence that regenerating RGC axons formed connections with the existing brain circuitry. Lastly, by using light pulse stimulation of the injured eye, c-Fos gene expression was observed in the SCN after the treatment, indicating functional synapses reformed in the SCN. Moreover, whole-cell patch-clamp recordings of SCN neurons with optic nerve stimulation showed evoked EPSCs, confirming reformed excitatory synaptic connections. Based on the same rationale, another recent study (Bei et al., 2016) adopted an optic tract transection model (proximal to the SC) that markedly reduced the distance between regenerating axons and the SC. In this study, either the combination of Pten/Socs3 co-deletion with CNTF/BCL2 or co-overexpression of OPN/IGF1/CNTF, induced retinocollicular axon regeneration and functional synapse formation after optic tract transection. However, regenerating axons exhibited poor electrical conduction and thus failed to restore significant visual function. One potential reason for the failure is likely associated with a lack of myelination. Voltage-gated potassium channel blockers (4-AP or 4-AP-3-Me) has been used to improve conduction in de-myelinated axons of patients with multiple sclerosis. As expected, acute application of 4-AP significantly enhanced the electrical conduction, eventually resulting in significant recovery of visual function. Together, these two studies with axon injury near their innervating targets provided key evidence that when sufficient regenerating axons could reach their brain targets synapse formation and visual function restoration are possible.

**Figure 2:**
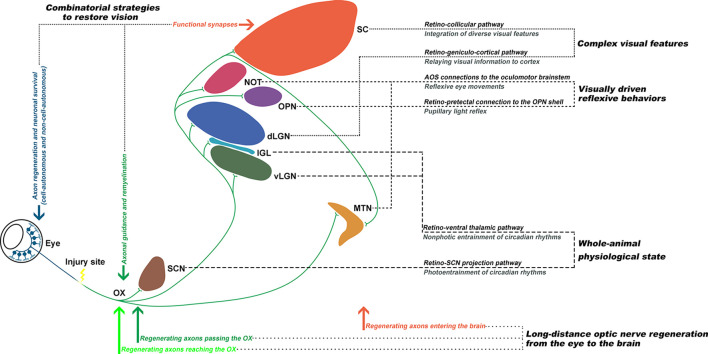
Functionally rewiring the eye-to-brain connections. The first step of an ideal repair strategy should be promoting sufficient long-distance regeneration of injured retinal ganglion cell (RGC) axons back to their original targets. Second, the regenerating axons need to be properly guided through the optic chiasm (OX) and reach their original innervating targets in the brain, which each mediates different visual functions. Third, for functional recovery, the regenerating axons need to reform functional synapses with the appropriate targets and remyelinate for electrical conduction. Finally, the optic nerve circuitries, governing the whole-animal physiological state, visually-driven reflexive behaviors, and complex visual features, could be re-established to restore visual functions.

In summary, the existing data up to date support that: (1) with combinatorial approaches, it is possible to induce long-distance axon regeneration to enter the brain; and (2) with proper axon guidance and remyelination, visual function could be restored (Figure 2). The eye-to-brain pathway contains multiple target structures, which are associated with various visual related functions, such as the whole-animal physiological state, visually-driven reflexive behaviors, and encoding complex visual features (Dhande et al., 2015). Therefore, the optimal solution for functional recovery after optic nerve injury requires long-distance axon regeneration from the injury site into the brain, proper axon guidance to reach specific central nuclei, reformation of functional synapses with the appropriate targets, and remyelination to enable transduction of electrical impulses.

## Future Prospects

Despite significant progress in RGC regeneration over the past decade, functional repair in the visual pathway still has a long way to go. Based on the above-described studies, an important question is where we should go soon. First, although we have identified many genes that can be manipulated to enhance optic nerve regeneration, our understanding of cellular and molecular mechanisms by which axon regeneration is regulated remain fragmented. For instance, during neuronal maturation what are the key steps and essential regulators that gradually switch off the ability of axon growth? What are the key differences between neurons in the CNS that almost permanently lost their capacity to support axon regeneration, comparing to those in the PNS that can reactivate their intrinsic capacity? Of all the neurons in the same tissue, do they all have the same ability to support axon regeneration? How do other cells in the retina, such as Müller cells, amacrine cells, contribute to the loss of axon regeneration ability of RGCs during maturation and failed regeneration after injuries? Recently developed multiomics approach, such as RNA-, ATAC, and Hi-C sequencing, either at bulk or single-cell level, supported by the advanced bioinformatics analyses, will be very useful tools to address these questions (Tran et al., 2019). On the other hand, the rapid updated CRISPR/CAS9-dCAS9 systems (Liu et al., 2018; Tian et al., 2019) are becoming mature and reliable techniques, which can be used for high-throughput functional screen of novel genes regulating RGC survival and regeneration. The application of these new techniques will not only help us better elucidate the molecular mechanisms underlying axon regeneration but also guide us to discover novel genes and pathways regulating RGC survival and regeneration.

Second, after long-distance axon regeneration, the next challenge is to guide the regenerating axons to reach their specific targets in the brain, form functional synapses, remyelinate the proper axons, and regain specific visual function. Unfortunately, up to date research in this area remains very limited. Although a few studies have shown long-distance regeneration of optic nerve axons back to all the brain nuclei and some visual function recovery, more studies are needed to confirm these findings. Several previous studies (Luo et al., 2013; Yungher et al., 2015; Wang et al., 2020) have shown clearly that high-resolution 3D imaging at the single axon level is an optimal approach to follow the trajectories of regenerating axons in the brain.

Third, the current most used animal model of visual injury is the optic nerve crush that is surgically easy and reproducible. However, multiple clinically relevant diseases result in optic nerve injury, such as glaucoma, optic neuritis, optic neuropathy, and optic nerve atrophy. Because different diseases may induce different cellular responses, different animal models mimicking each disease should be utilized before a potential translational application.

## Author Contributions

S-GY, C-ML, and F-QZ designed, wrote, revised, and finalized the manuscript. C-PL edited the manuscript. X-QP designed the figures. Z-QT provided constructive comments. All authors read and approved the final version of the manuscript.

## Conflict of Interest

The authors declare that the research was conducted in the absence of any commercial or financial relationships that could be construed as a potential conflict of interest.
